# Doxapram alleviates low SpO_2_ induced by the combination of propofol and fentanyl during painless gastrointestinal endoscopy

**DOI:** 10.1186/s12871-019-0860-1

**Published:** 2019-11-22

**Authors:** Zhengfeng Gu, Lian Xin, Haoxing Wang, Chunxiao Hu, Zhiping Wang, Shunmei Lu, Jingjing Xu, Yiling Qian, Jun Wang

**Affiliations:** Department of Anesthesiology, Wuxi People’s Hospital Affiliated to Nanjing Medical University, 299 Qingyang Road, Wuxi, 214023 Jiangsu China

**Keywords:** Propofol, Doxapram, Respiratory depression, Painless gastrointestinal endoscopy, MAP, HR, SpO_2_

## Abstract

**Background:**

Painless gastrointestinal endoscopy under intravenous propofol anesthesia is widely applied in the clinical scenario. Despite the good sedation and elimination of anxiety that propofol provides, low SpO_2_ may also result. Doxapram is a respiratory stimulant with a short half-life. The primary aim of this study was to investigate the effects of doxapram on alleviating low SpO_2_ induced by the combination of propofol and fentanyl during painless gastrointestinal endoscopy.

**Methods:**

In this prospective study, patients scheduled for painless gastrointestinal endoscopy were randomly assigned to group D or S with 55 patients per group. Initially, both groups received a combination of propofol and fentanyl. Patients in group D received 50 mg doxapram after propofol injection, while patients in group S received an equal volume of saline. Vital signs of the patients, propofol dose, examination duration, and incidences of low SpO_2_ were recorded.

**Results:**

There were no statistical differences in propofol consumption and examination duration between the two groups. Twenty-six patients in group S experienced low SpO_2_ versus 10 in group D (*P* = 0.001). Nineteen patients in group S underwent oxygenation with a face mask in contrast to 8 in group D (*P* = 0.015). Eighteen patients in group S were treated with jaw lifting compared to 5 in group D (*P* = 0.002). Four patients in group S underwent assisted respiration compared to 2 in group D (without statistical difference). The average oxygen saturation in group S was significantly lower than that in group D at 1, 2 and 3 min after propofol injection (*P* < 0.001, *P* = 0.001 and *P* = 0.020, respectively). There were no statistical differences in oxygen saturation at other time points. There were no statistical differences in MAP and HR (except for the time point of 1 min after the induction) between the two groups.

**Conclusions:**

Low dose of doxapram can effectively alleviate low SpO_2_ in painless gastrointestinal endoscopy with intravenous propofol, without affecting propofol consumption, examination duration, MAP, or HR.

**Trail registration:**

The study was approved by the Institutional Ethics Committee of Clinical and New Technology of Wuxi People’s Hospital on 20th July, 2018 (KYLLH2018029) and registered in the Chinese Clinical Trial Register on 16th August, 2018 (ChiCTR1800017832).

## Background

Painless gastrointestinal endoscopy is increasingly applied in Class-A Tertiary Hospitals in China. Many patients prefer painless gastrointestinal endoscopy under sedation with analgesia for the pain resulting from mesenteric traction maneuvers, colonic distension by gas insufflations, and winding of the device within the intestinal tract. Accordingly, propofol, combined with analgesics, is commonly used for these procedures [1–3]. This method reduces anxiety and discomfort, improves tolerability and patient satisfaction, and provides better effects for the procedure.

The advantages of propofol include earlier onset, shorter examination duration and quicker emergence. However, in the case of intravenous administration, low SpO_2_ and circulatory inhibition may occur. Low SpO_2_ resulting from propofol, especially in combination with an analgesic, such as fentanyl, can potentially render risk to patients undergoing painless gastrointestinal endoscopy, in which case anesthesia with intravenous administration of propofol requires rigorous supervision by an experienced anesthesiologist. Doxapram is a respiratory stimulant with a short duration and fast onset [4]. Low-dose doxapram may excite the respiratory center by stimulating the chemoreceptor of the carotid sinus, whereas large dose of doxapram can directly excite medullar respiratory center, spinal cord and brainstem, which could lead to increased tidal volume [5]. As per the pharmacological mechanism, we postulated that doxapram should well alleviate low SpO_2_ induced by propofol during painless gastrointestinal endoscopy, without affecting the quality of anesthesia. The primary aim of this study was to investigate the effects of doxapram on alleviating low SpO_2_ induced by the combination of propofol and fentanyl during painless gastrointestinal endoscopy. Our prospective study specifically compared the administration of propofol and fentanyl with or without doxapram for painless gastrointestinal endoscopy performed by anesthesiologists.

## Methods

Patients scheduled for painless gastrointestinal endoscopy were recruited for this prospective, randomized, double-blind study. The trial was conducted at the Department of Gastroenterology, Wuxi People’s Hospital from August 2018 through January 2019. It was approved by the institutional Ethics Committee of Clinical and New Technology of Wuxi People’s Hospital (KYLLH2018029) and registered in the Chinese Clinical Trial Register (ChiCTR) (ChiCTR1800017832). The trial mainly aimed to evaluate the effects of doxapram on propofol-induced low SpO_2_ when combined with fentanyl analgesia. Secondly, we evaluated the total consumption of propofol, examination duration, mean arterial pressure (MAP), and heart rate (HR). The study adheres to consolidated standards of reporting trials.

All patients over 18 years of age scheduled for a diagnostic gastrointestinal endoscopy were included in this study, after submission of written informed consent. The exclusion criteria included medical history such as medication of diazepam, neuroleptics, and anticonvulsants that interfere with heart rate; anaphylaxis to drugs used in the study; cardiovascular diseases such as hypertension, arrhythmia, abnormal electrocardiogram (ECG); abnormal liver and/or kidney functions; lung disease, such as chronic obstructive pulmonary disease (COPD); abdominal laparotomy; body mass index above 30 kg·m^− 2^; age over 75 years or below 18 years; clinical suspicion of intestinal subocclusion or stenosis; colorectal tumors; psychiatric patients; and requirement for complex therapeutic procedures during diagnostic colonoscopy.

Sample size calculation was performed with a probability of type I error (α) at 0.05, a power (1-β) of 0.90, a low SpO_2_ of 80 and 50% in the control and intervention groups, respectively. Thus, 52 patients were required in each group. Considering an approximately 5% loss to follow-up, we included 55 patients in each group.

An anesthetist nurse prepared the same volume of doxapram or saline as per the randomization by the computer in the envelope. Both the patients and the anesthesiologists in charge of anesthesia were blinded to the allocation. All patients were monitored with pulse oximetry, continuous ECG, and noninvasive blood pressure assessed every 1 min in the first 5 min and then at a 5-min interval after doxapram or saline administration. The outcome was assessed and recorded by another anesthetist nurse who was blinded to study group assignment. Each patient in Group D received intravenous infusion (IV) of fentanyl 0.05 mg and propofol 1–2 mg·kg^− 1^ sequentially, followed by IV doxapram 50 mg, and patients in Group S individually received sequential IV fentanyl 0.05 mg and propofol 1–2 mg·kg^− 1^, followed by IV saline (same volume as doxapram in group D). In both groups, anesthesia was induced with propofol 1–2 min after IV fentanyl, and the total dose was slowly infused within 60 s, or limited to the drooping eyelid with loss of corneal-palpebral reflex (Ramsay Score 6). An additional dose of 0.5 mg·kg^− 1^ was given in the event of signs of motor function. In both groups, oxygenation was applied with a nasal tube (3 L/min). Gastrointestinal endoscopy was performed by the same endoscopist using an Olympus OEV262H video system with gastroscopic tubes from the GIF-H290 series and colonoscopic tubes from the CF-H2901 series. The endoscopists performed gastroscopy and colonoscopy sequentially.

The primary aim of the study was to investigate the effects of doxapram on low SpO_2_. Low SpO_2_ was considered significant when SpO_2_ was < 90% [6]. A face mask covering the patient’s nose and mouth was applied when the SpO_2_ < 90%. The method of jaw lifting would be applicable when the SpO_2_ was still less than 90% 10 s after face mask application. Assisted ventilation with a simple breathing balloon would be performed immediately in the case of the SpO_2_ still below 90% 10 s after jaw lifting.

Patients were transferred to the post-anesthesia care unit (PACU) under the care of an experienced anesthetist nurse on completion of the endoscopy. After 30 min in the PACU, we evaluated the satisfaction scores of the endoscopists and patients using a visual analog scale (VAS, 0 = dissatisfaction, and 10 = full satisfaction). Patients were considered eligible for discharge with a score ≥ 9, according to the modified Aldrete–Kroulik index [7].

Statistical analyses were performed using Medcalc software (version 15, Medcalc Software bvba, Ostend, Belgium) [8]. Age, weight, and height of patients, total examination duration, total propofol consumption, BP, HR, and SpO_2_ were recorded, together with profiles as low SpO_2_, face mask use, jaw lifting, and assisted ventilation. Study outcomes included the development of low SpO_2_ (< 90%) and the necessity of the following managements by minutes. The variations of MAP and HR were compared as well as the satisfaction of both endoscopists and patients. The gender proportions and cases of low SpO_2_ were compared with a Pearson Chi-squared test. Levels of outcome parameters were compared with independent samples *t*-test after checking for normal distribution. Mann-Whitney test (independent samples) was employed if the parameters presented an abnormal distribution. *P* < 0.05 was considered statistically significant.

## Results

A total of 110 patients rated as ASA I-II were enrolled in this study. All patients underwent a complete gastrointestinal endoscopy. Table 1 showed study data with no statistical differences, with respect to gender, age, weight, and height. The dosages of propofol consumption, examination duration, and satisfaction VAS of endoscopists and patients were also similar between the two groups.

**Table 1:** Demographics, propofol, examination duration, and satisfaction of VAS

Parameter	Group D (*n* = 55)	Group S (*n* = 55)	Statistics	*P*
Gender (f/m)	33/22	30/25	1.200	0.273
Age (yr)	46.2 ± 11.50	50.0 ± 10.10	1.839	0.069
Weight (kg)	60.8 ± 10.10	63.0 ± 10.10	1.107	0.271
Height (cm)	164.1 ± 7.40	165.5 ± 8.10	0.936	0.351
BMI (kg·m^−2^)	22.5 ± 2.80	22.9 ± 2.30	0.788	0.433
Propofol (mg)	262.3 ± 53.70	244.0 ± 60.60	−1.681	0.096
Duration (s)	810.1 ± 243.60	781.6 ± 284.60	−0.566	0.573
VAS (endoscopist)	9.7 ± 0.20	9.6 ± 0.20	−0.130	0.898
VAS (patient)	9.8 ± 0.20	9.8 ± 0.20	0.170	0.866

Note: data presented as mean ± standard deviation (SD)

**Fig. 1:**
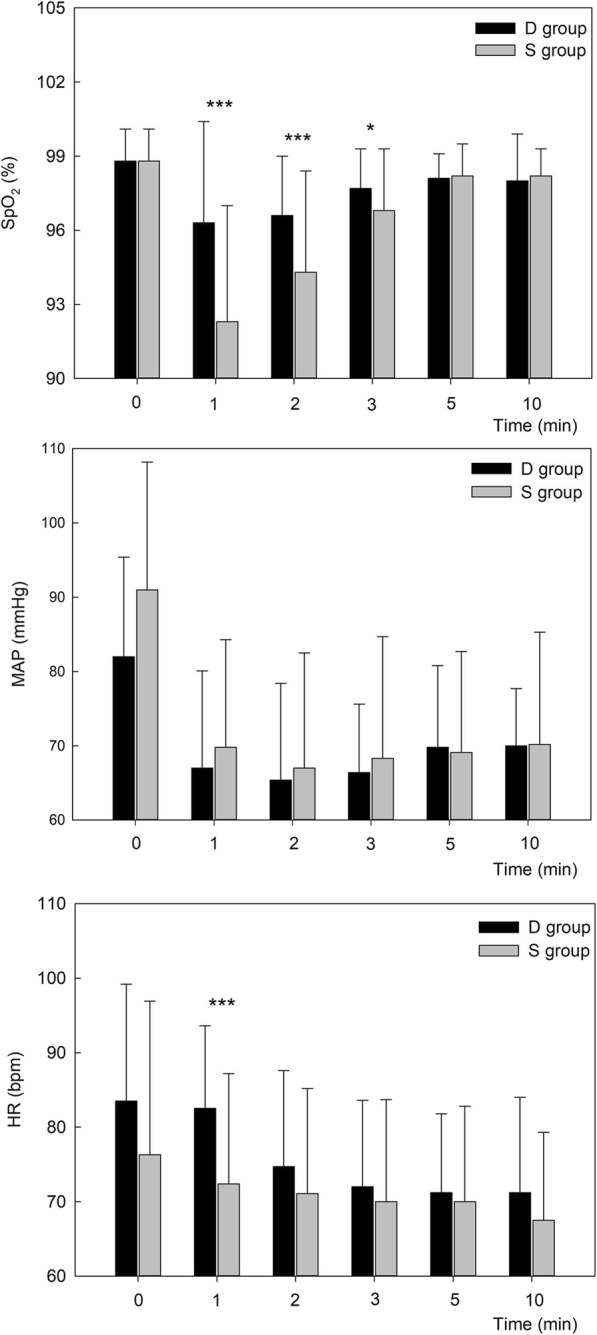
Variation of SpO_2_, MAP, and HR between group D and group S at different time points (*n* = 55 per group). Note: data are presented as mean ± standard deviation (SD). * represents significant differences at *P* < 0.05

As shown in Fig. 1, SpO_2_ was significantly higher in group D than in group S at 1, 2, and 3 min after propofol injection (*P* < 0.001, *P* = 0.007, and *P* = 0.020, respectively). There were no statistical differences in SpO_2_ at other time points (Fig. 1). MAP decreased after propofol administration in both groups. There were no statistical differences in MAP at any time point between the two groups. HR measurements, at 1 min after propofol infusion, were significantly lower in group S compared to group D (72.4 ± 14.8 vs. 82.5 ± 11.1, *P* = 0.001). No other side effects of doxapram were observed throughout the experiment.

Patients in group S had a higher incidence of low SpO_2_ compared to those in group D (*P* = 0.001), as shown in Table 2. There were significantly lower incidences of face mask use and jaw lifting in group D compared to those in group S (*P* = 0.015 and *P* = 0.002, respectively). Four patients in group S and 2 in group D underwent assisted ventilation, with insignificant statistical difference.

**Table 2:** Cases of respiratory depression and therapy

	S (*n* = 55)	D (*n* = 55)	*χ*^2^	*P*
Low SpO_2_	26	10	10.5706	0.001*
Inhaling of oxygen with a face mask	19	8	5.9393	0.015*
Jaw lifting	18	5	9.2904	0.002*
Assisted ventilation	4	2	0.7051	0.401

* represents significant differences at *P* < 0.05

## Discussion

Gastrointestinal endoscopy is a common procedure for prevention, diagnosis, and treatment of a variety of symptoms and diseases of the stomach and lower digestive tract. Sedation or anesthesia is an important means to increase comfort and decrease anxiety, discomfort and pain during the endoscopic maneuver [9]. Propofol is a satisfactory intravenous anesthetic due to its short half-life, fast emergence from anesthesia, and low incidence of nausea and vomiting. It is widely used in outpatient gastrointestinal endoscopic procedures for sedation and/ or anesthesia without increasing cardiopulmonary adverse events compared to conventional agents [10]. It has a very narrow therapeutic window, which can easily progress from moderate to deep sedation or general anesthesia without a reversal agent [11]. Thus, anesthetists must be meticulous for the side effects of propofol, including injection pain, hypotension, bradycardia, and low SpO_2_ [12]. In our study, the administration of intravenous propofol combined with fentanyl, as the anesthetic agent, resulted in a high incidence of low SpO_2_ (34.5%). Despite the respiration-assisted techniques and airway management available to ensure patients’ safety, decreasing the incidence of low SpO_2_ without affecting the quality of anesthesia is of interest to clinicians [13]. Intravenous propofol is routinely combined with a small dose of fentanyl and/or midazolam to assist in sedation and analgesia during colonoscopies on the grounds of its short analgesic effects [14–16]. Fentanyl, a synthetic opioid analgesic, has good analgesic effects at small doses. It is fast-acting and results in fewer low SpO_2._ Fentanyl is often administered in outpatient painless colonoscopy in combination with propofol to reduce propofol consumption, promote emergence and abridge theater stay. Unfortunately, both propofol and fentanyl may result in low SpO_2_. Thus, the combined application may render patients at increased risk of low SpO_2_. On the contrary, doxapram, a fast and short-acting respiratory stimulant, may reduce the incidence of low SpO_2_ from propofol and fentanyl [17], and is frequently applicable to low SpO_2_ due to anesthesia or central inhibition [18]. Moreover, doxapram can also serve as an analeptic after general anesthesia, such as sevoflurane inhalation [19].

Our results revealed that SpO_2_ was significantly increased at 1, 2, and 3 min subsequent to propofol injection in the group treated with doxapram compared to the saline-treated group. We also observed a decreased incidence of low SpO_2_, oxygen inhalation with a face mask and jaw lifting in the group treated with doxapram compared to the saline-treated group. These outcomes may be related to the effect of respiratory stimulation of doxapram and its action duration. Doxapram can reflexively stimulate the respiratory center via chemical receptors in the carotid body at low doses. At large doses, however, doxapram directly stimulates the respiratory center in the medulla oblongata [20]. S. Kruszynski et al. [21] attributed that part of the stimulatory effects of doxapram to the direct input on brainstem centers with differential effects on the rhythm generating kernel (PreBötzinger Complex) and the downstream motor output. Propofol acts as an agonist of γ-aminobutyric acid (GABA) receptor. GABA_B_ activates the channel of K^+^ on postsynaptic membrane and leads to hyperpolarization of the latter. Doxapram blocks the K^+^ channel on postsynaptic membrane via Ca^2+^-dependent K^+^ conductance [22]. We speculated that this action might be one of the mechanisms underlying doxapram antagonizing the side effect of propofol. Our study demonstrated that doxapram could effectively alleviate the occurrence of low SpO_2_ during gastrointestinal endoscopy, thus providing improved safety of patients.

Despite the alleviation of low SpO_2_ by doxapram, precautions should be taken against the side effects whereby. Doxapram may induce headache, dyspnea, arrhythmia, diarrhea, nausea, vomiting, chest pain, and hypertension, etc., among which arrhythmia, dyspnea and hypertension are the most relevant and severe side effects. Notwithstanding the absence of adverse effects as arrhythmia, dyspnea and hypertension in this study, our small sample size did not suffice to reach a compelling conclusion for a risk-benefit balance of doxapram. Consequently, larger trials are required in order to verify the definite role of doxapram during general anesthesia for gastrointestinal endoscopy.

The development of hypotension and bradycardia in anesthesia is probably attributable to the effects of vascular dilation and myocardial inhibition by propofol on the gamma-aminobutyric acid receptors and the atrial muscarinic cholinergic receptors [23]. Doxapram causes transient tachycardia (1 min after injection), which may be related to the stimulation of catecholamine release via β_1_-receptor stimulation. In our study, even in the event of hypotension and bradycardia, doxapram may not have adequately exerted its reversive effects largely due to the relatively low dosage.

Doxapram is occasionally applied to promote emergence from volatile anesthesia, such as sevoflurane [19]. Our findings demonstrated that regardless of doxapram administration, all the 110 patients were discharged with a modified Aldrete–Kroulik index > 9 within 30 min, which indicated that doxapram did not affect the time required for emergence from anesthesia. We speculated that the inefficacy on emergence timing was due to the doxapram administered at the commencement rather than the denouement of anesthesia, as in the study by HL Wang et al. using total intravenous anesthesia with dexmedetomidine, propofol and remifentanil [24].

There are some limitations in our study. First, our results would have been more accurate provided that propofol delivery had been guided by the monitoring of anesthetic depth, as with a bispectral index for instance. Despite the same dose of doxapram we adopted for each patient, questions still remained as to whether the dose should be administered as per body weight. Second, we did not monitor ETCO_2_ as an element of respiratory depression and moreover we did not increase the flow rate of oxygen prior to application of face mask/jaw lifting or ventilation with constant flow rate for each patient, either. Furthermore, we did not employ SpO_2_/FiO_2_ ratio to evaluate respiratory depression, and we did not monitor for postoperative pulmonary complications for the patients in PACU. Nonetheless, with respect to the care of outpatients, early safe discharge from PACU is of importance for the improved medical efficiency and the medical care system at large, thus awaiting more profound investigations as to whether doxapram could decrease the emergence time in scenario of PACU.

## Conclusion

With the addition of 50 mg of doxapram in intravenous anesthesia with propofol and fentanyl for gastrointestinal endoscopy, the incidence of hypoxemia and the necessity of respiratory assistance following anesthetic induction were significantly reduced for the initial 3 min. The medication of doxapram did not affect the satisfaction scores of the endoscopists and has little effect on MAP and HR at the 50 mg dose.

## Data Availability

All data generated or analyzed during this study are included in this published article and are available from the corresponding author on reasonable request. The trial profile is available at http://www.chictr.org.cn.

## Abbreviations

HR: Heart rate
IV: Intravenous injection
MAP: Mean arterial pressure
PACU: Post anesthesia care unit
SpO2: Oxygen saturation
VAS: Visual analogue scale
